# The relationship between amyloid structure and cytotoxicity

**DOI:** 10.4161/pri.28860

**Published:** 2014-05-12

**Authors:** Karen E Marshall, Ricardo Marchante, Wei-Feng Xue, Louise C Serpell

**Affiliations:** 1 School of Life Sciences; University of Sussex; Falmer, East Sussex UK; 2 School of Biological Sciences; University of Kent; Canterbury, Kent UK

**Keywords:** amyloid, amyloidosis, cross-β, cytotoxicity, deposition, fibril, oligomer, structure

## Abstract

Self-assembly of proteins and peptides into amyloid structures has been the subject of intense and focused research due to their association with neurodegenerative, age-related human diseases and transmissible prion diseases in humans and mammals. Of the disease associated amyloid assemblies, a diverse array of species, ranging from small oligomeric assembly intermediates to fibrillar structures, have been shown to have toxic potential. Equally, a range of species formed by the same disease associated amyloid sequences have been found to be relatively benign under comparable monomer equivalent concentrations and conditions. In recent years, an increasing number of functional amyloid systems have also been found. These developments show that not all amyloid structures are generically toxic to cells. Given these observations, it is important to understand why amyloid structures may encode such varied toxic potential despite sharing a common core molecular architecture. Here, we discuss possible links between different aspects of amyloidogenic structures and assembly mechanisms with their varied functional effects. We propose testable hypotheses for the relationship between amyloid structure and its toxic potential in the context of recent reports on amyloid sequence, structure, and toxicity relationships.

## From Sequence to Amyloid Structure

Amyloid fibrils are highly organized and stable structures composed of proteins that are able to self-assemble via β-sheet association. The core of all amyloid fibrils adopt what is commonly known as the cross-β structure, named due to the arrangement in which β-strands that run perpendicular to the fiber axis form β-sheet ribbons or filaments that further associate to form amyloid fibrils.^1^ These fibrils are found in a large number of protein misfolding diseases including Alzheimer disease, type 2 diabetes mellitus, and transmissible spongiform encephalopathies.^2^ Peptides and proteins with different sequences are able to readily form amyloid-like fibrils. Thus, the sequence determinants of amyloid self-assembly have been an area of intense research. Algorithms have been developed to analyze amino acid sequences and to predict their propensity to form amyloid. These have used properties such as β-sheet propensity and hydrophobicity^3^^,^^4^ or more complex methods to look at the relationship between the arrangement of the amino acids and the final cross-β structure.^5^^,^^6^ From these studies, it has become clear that many short peptides have a high propensity to form amyloid fibrils^6^; over 50 have been identified to date and the number is growing. Recent advances in characterizing the structural properties of amyloid fibrils show an increasingly detailed atomic resolution picture of the cross-β structure. X-ray crystallography provides structural information, showing that small amyloid forming peptides are able to form steric zippers in which side chains associate and interlock between the β-sheets.^7^ X-ray fiber diffraction^8^^,^^9^ and solid-state NMR^10^^-^^12^ have been used to examine the structure of the fibrils themselves, and show similar complementarity between the side chain interactions across the sheets. These observations further anchor the notion of a similar core structural organization for all amyloid fibrils (Fig. 1A–C).

**Figure:**
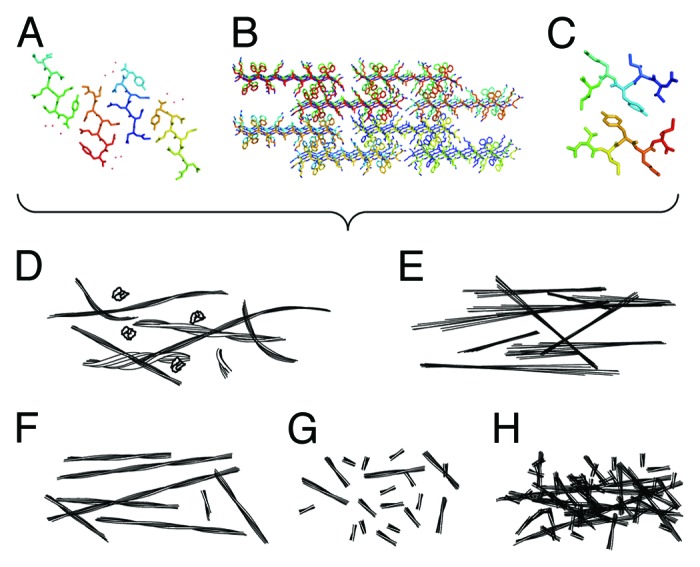
**Figure 1.** Detailed atomic resolution structures of amyloid leads to different physical properties, which in turn produce varied biological functions. (**A–C**) Structural models depicting the amyloid cross-β molecular architecture. (**A**) the crystal structure of GNNQQNY (2OMM.pdb),^7^ (**B**) a model of fibrous nanocrystals formed by KFFEAAAKKFFE,^8^ (**C**) a model structure of fibrils formed by VIYKI.^9^ (**D–H**) schematic illustrations of morphologies that may result in different biological properties. (**D**) Illustrations of flexible and heterogeneous fibrils compared with (**E**) rigid and crystal-like fibrils. (**F**) Illustrations showing long and thermally, mechanically and/or enzymatically stable fibrils compared with (**G**) short, fragile, and unstable fibrils with non-interacting surfaces and (**H**) fibrils with interacting or sticky surfaces.

## Toxic and Functional Properties of Amyloid

In amyloid associated disease, amyloid fibrils are deposited in tissues, forming insoluble structures and sometimes replacing cells. For example, in lysozyme amyloidosis, much of the kidney can be replaced by amyloid deposits.^13^ However, some small oligomeric intermediates formed during the self-assembly by amyloidogenic precursor proteins or peptides have toxic potential and are involved in the cell death observed in these misfolding and neurodegenerative diseases,^14^ although not all small oligomeric intermediates have been found to be toxic. For example, HypF can be induced to form both toxic and non-toxic oligomeric species.^15^ Equally, small fibrillar oligomer species or fibril fragments have also been found to possess toxic potential.^16^^-^^18^ Indeed, a β-sheet protein that has been designed to form amyloid-like oligomers leads to a cascade of toxic events following expression in human cells.^17^ Recently, it has become clear that amyloid structures are quite commonly found in natural systems, and there are increasing reports of such functional amyloid structures that provide scaffold, support, and protection due to their stability and strength.^19^ For example, *E. coli* produces curli that forms a layer of amyloid fibrils on the outside of the organism,^20^ while amyloid has also been identified as forming a protective layer to melanosomes.^21^ Interestingly, the self-assembly of each of these systems is carefully controlled by the organism,^22^ although whether such functional amyloid assemblies inherently lack toxic potential remains to be established.

These observations together with recent advances in the determination of the sequence and structural properties of amyloid fibrils raise an important question: Why are some amyloid assemblies toxic, while others are inert or tolerated, even to the extent that they may be evolved to perform useful biological functions, despite the fact that the end products of amyloid assembly share a common cross-β structural organization?

## In Search of Structure and Assembly Properties that Confer Toxic Potential

The varied toxic potential of amyloid assemblies may involve several structural and assembly properties such as different levels of structural rigidity, stability (thermal, enzymatic, or mechanical), and surface protection of amyloid fibrils (Fig. 1D–H). These form testable hypotheses that can be examined by studies that connect sequence, structure, morphology, and toxicity of amyloid assemblies.

Some short amyloidogenic peptides identified by algorithms and crystallography, are some of the most efficient and effective amyloid fibril formers. They self-assemble rapidly and effectively, forming highly organized and rigid fibrils and sometimes crystal-like aggregates^23^ (Fig. 2). Using these highly effective self-assembling peptides, we investigated the cytotoxic properties of some of these rigid crystal-like assemblies in neuroblastoma cell-line cultures, compared with toxic, oligomeric Aβ(1−42) (Aβ_42_) (Fig. 3). We investigated the effect of soluble peptide as well as crystalline and fibrillar aggregates formed by the peptide KFFEAAAKKFFE and variants of this sequence.^24^ Interestingly, none affected the reduction of MTT to formazan, a process that occurs in viable cells with active metabolism, suggesting that these aggregates possess little cytotoxic potential. For comparison, it is well known that Aβ, in various forms, is toxic to cells. Aβ peptides self-assemble rapidly,^25^ particularly Aβ_42_. However, Aβ_42_ does form significant populations of potentially long-lived and toxic intermediates that can be visualized by electron microscopy.^16^^,^^26^ Aβ also has the potential to form a wide range of different species and fibril polymorphs.^27^^,^^28^ Thus, the lack of structural rigidity in the fibrillar assembly products of Aβ amyloid may reflect the ability of Aβ to participate in heterogeneous assembly reactions that could form a large number of different intermediate and fibril species, some of which may possess cytotoxic potential.

**Figure:**
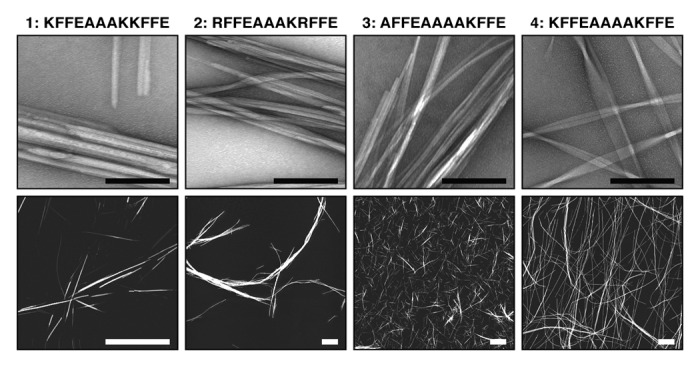
**Figure 2.** Structure and morphology of different amyloid aggregates assembled from KFFEAAAKKFFE ^8^ and variants ^24^ results in altered morphology and assembly properties. Images show aggregated peptide samples 1: KFFEAAAKKFFE in PBS, 2: RFFEAAAKRFFE in PBS, 3: AFFEAAAAKFFE in water, and 4: KFFEAAAAKFFE in water. Upper row shows negative stain transmission electron microscope images (black scale bars = 200 nm). Lower row shows atomic force microscopy height images (white scale bars = 2 μm).

**Figure:**
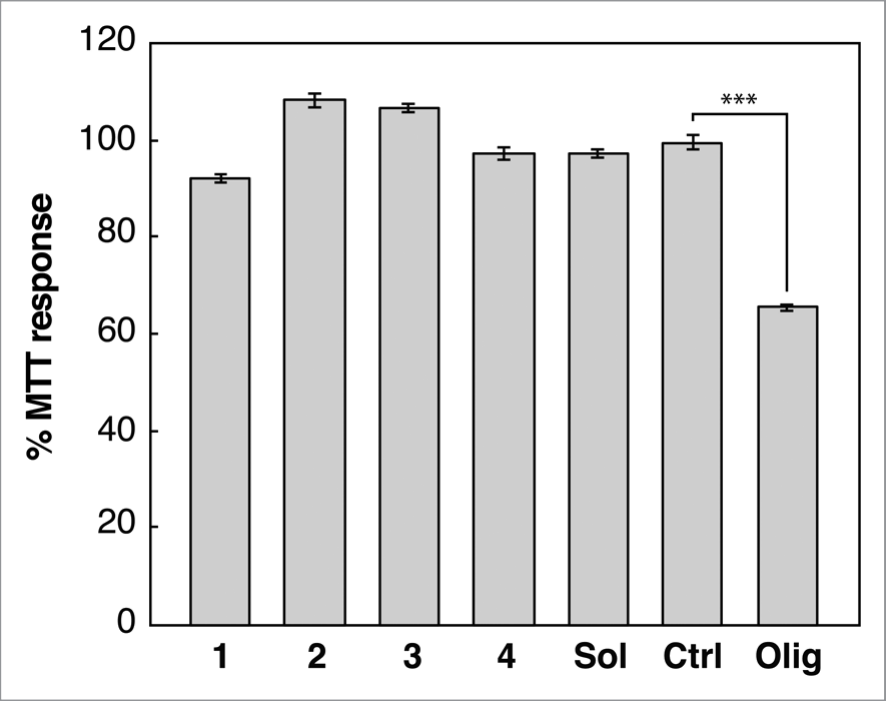
**Figure 3.** The effect of assembled peptide morphology and composition on neuroblastoma cells. MTT assays (Molecular Probes) were performed according to the manufacturers protocol. A neuroblastoma cell line (SH-SY5Y) was treated with 10 μM monomer equivalent concentrations of aggregated peptide samples 1: KFFEAAAKKFFE in PBS 2: RFFEAAAKRFFE in PBS, 3: AFFEAAAAKFFE in water, and 4: KFFEAAAAKFFE in water (Fig. 2). Cells were also treated with soluble KFFEAAAKKFFE in water (labeled Sol), a buffer only control (labeled Ctrl) or Aβ_42_ oligomers (labeled Olig). Changes in the MTT response after 24 h were monitored and are shown relative to the buffer control and error bars correspond to SEM over 3 replicates. Only Aβ_42_ oligomers^16^^,^^26^ induce cellular dysfunction resulting in a reduction to 60% compared with control. The student *t* test comparison between olig and ctrl showed significance of*P* < 0.001 labeled ***.

Another property to consider is the stability of amyloid fibrils, and their reversibility and disassembly processes. The assembly process of amyloid is reversible, and this dynamic process is normally favored toward the formation of amyloid fibrils. Thus, fibrils that are thermodynamically and/or kinetically more stable will disassemble more slowly and, therefore, shed a lower population of potentially cytotoxic species^29^ from fibril ends at a lower rate. The mechanical stability of amyloid fibrils may also play a key role in generating small fibril fragments (or fibrillar oligomers) that possess cytotoxic potential through fibril breakage processes induced by mechanical or enzymatic action.^30^^,^^31^ Thus, understanding the origin of the toxic properties of amyloid assemblies will involve the characterization of the stability of fibrils toward different perturbations, and the sequence and structural properties that confer the different stabilities of fibrils.

The surface properties of amyloid fibrils may also influence their toxic potential. For example, small oligomeric species and fibrillar fragments have all been shown to be able to disrupt lipid membranes.^18^^,^^32^^,^^33^ For fibril fragments, fibril ends have been seen as the active surfaces that interact with membranes.^32^^-^^34^ Thus, the lack of surface protection that can minimize aberrant interactions at the sites of amyloid growth may play an important role in terms of their cytotoxic potential. Such protection, for example in the form of a mechanism involving capping of growth competent fibril ends, may also be a generic feature that provides a host organism the means to control functional amyloid. Surfaces that promote fibril-fibril interactions, or the packing between proto-filaments in fibrils may further modulate the reactivity and stability of amyloid fibrils, deposits, or plaques, in turn affecting the generation of potentially toxic species through depolymerisation. Detailed atomic resolution structural information of amyloid fibrils may inform on their surface properties, for example revealing hydrophobic patches that may act as “sticky” sites that can promote aberrant interactions or fibril clumping.

## Amyloid by Design

Finally, a large area of synthetic biology has grown from the use of β-sheet structural growth, providing nanowires and hydrogels composed of amyloid-like structures.^11^^,^^35^^,^^36^ The amyloid structure may prove to be useful and may be exploited to form a range of materials.^37^ These fibrils are formed by repetitive precursor sequences, often short peptides, that form highly organized fibrils. These can be decorated further, or they can be used to form hydrogels or wires.^38^^,^^39^ β-sheet based materials have been used in tissue engineering and for enabling cell growth, showing they are non-toxic.^35^

Ultimately, engineering synthetic amyloid as a novel and safe bionanomaterial will rely on the understanding of the structural factors that underpin toxic properties so that the toxic potential of designed amyloid materials can be minimized or avoided. In the same way, understanding the same underlying principles will be key in understanding amyloid disease so that the toxic potential of disease associated amyloid can be inhibited.

## Notes

MorrisKLRodgerAHicksMRDebulpaepMSchymkowitzJRousseauFSerpellLC Exploring the sequence-structure relationship for amyloid peptides Biochem J 2013 450 275 83 10.1042/BJ20121773

MarshallKEMorrisKLCharltonDO’ReillyNLewisLWaldenHSerpellLC Hydrophobic, aromatic, and electrostatic interactions play a central role in amyloid fibril formation and stability Biochemistry 2011 50 2061 71 10.1021/bi101936c

## Notes

10.4161/pri.28860
